# Do participants in a physical activity program from a Care Sport Connector become healthier? An explorative study from the Netherlands

**DOI:** 10.1371/journal.pone.0287913

**Published:** 2023-12-14

**Authors:** E. Smit, K. E. F. Leenaars, M. A. E. Wagemakers, E. J. Bakker, J. van der Velden, G. R. M. Molleman

**Affiliations:** 1 Academic Collaborative Centre AMPHI, Primary and Community Care, Radboud University Medical Center, Nijmegen, The Netherlands; 2 Department of Social Sciences, Health and Society Group, Wageningen University & Research Centre, Wageningen, The Netherlands; University of Hertfordshire, UNITED KINGDOM

## Abstract

**Introduction:**

Care Sport Connectors (CSCs) have been appointed to create a connection between the primary care and physical activity (PA) sectors to stimulate residents who are inactive to become more physically active to gain health benefits. The objective of this explorative study was to find out whether CSCs achieve these goals by testing the hypothesis that more residents become physically active, and score higher for health-related fitness and health-related quality of life.

**Method:**

We conducted a longitudinal study design whereby participants (n = 402) were measured at three time points: at the start of their PA program (T_0_); after 6 months (T_1_); and after 1 year (T_2_). Participants conducted a fitness test to measure their health-related physical fitness and filled in questionnaires to assess PA level (PA-, Fit-, Combi-, and sport norm), health-related quality of life, motivation for PA, and personal information. We used a multi-level analysis to test whether outcomes of participants differ over time. Participants who dropped out and maintainers were compared with a chi-square test and a one-way ANOVA.

**Results:**

This study showed that one-third of the participants dropped out (n = 139). Participants who dropped out were, compared with maintainers, less physically active (P = 0.004) and were more often reached in bigger municipalities, by an integral approach. More participants meet the PA norm (P = 0.007) and sport norm (P<0.001) at T_2_ then at T_0_. Scores in health-related physical fitness and quality of life were significant but not a meaningful gain in health-related fitness.

**Conclusion:**

More residents become physically active and participate in sport because they took part in a PA programs or activity organized by a CSC. Lifestyle interventions should be offered with a higher frequency, intensity, and focus on behavior change. It is necessary to invest in combined lifestyle interventions offered by a collaboration of primary care, welfare, and PA professionals.

## Introduction

Physical activity (PA) has been recognized as an important determinant for health, and there is substantial evidence for using exercise as medicine for individuals with or at risk of chronic disease [1, 2]. However, it is challenging to influence PA behavior due to interrelated factors at multiple levels—individual, social, environmental, and policy—that influence lifestyle behaviors [3]. Therefore, an integrated approach is required to link the talents, resources, relationships, and approaches of different sectors and professions, and to affect all these interrelated factors more effectively, efficiently, and sustainably than one sector or profession could achieve alone [3–6].

In 2012, the Dutch Ministry of Health, Welfare and Sport introduced Neighborhood Sport Coaches (Buurtsportcoaches) to facilitate intersectoral collaboration in their role as a broker. They facilitate an integral approach at the community level to stimulate residents who are inactive to become more physically active. For this, the Neighborhood Sport Coach uses local partners and sources to create a sustainable local PA environment. They have been appointed in 98.6% of the municipalities in the Netherlands [7]. Additionally, the Ministry of Health, Welfare and Sport provides subsidies to local providers of sports and physical activities for the implementation of proven PA programs. In this way, local facilities are utilized, the offer can be tailored to the wishes and needs of the target group, and existing knowledge is used to stimulate residents who are inactive to become more physically active in order to lower the number of residents who have a chronic diseases [8].

People who are inactive and suffer from or have a high risk of developing a chronic disease are usually known by primary care professionals. Therefore, some Neighborhood Sports Coaches especially focus on the collaboration between the primary care and PA sectors: the so-called Care Sport Connectors (CSCs). The rational is that CSCs facilitate the connection between the primary care and PA sectors. Professionals from these sectors then collaborate and implement PA programs, which reach certain target groups. Eventually, these target groups will become able to self-manage their PA: they will become more physically active in their neighborhoods and their health outcomes will improve.

However, health is a broad concept and is related to various factors. Bouchard and Sheppard described these dependencies in the Toronto Model [9]. The model shows that physical activity (leisure, occupational, and other chores) can influence health related fitness (metabolic, morphological, muscular, motor, and cardiorespiratory), that subsequently affects health (wellness, morbidity, and mortality), but this is also possible in a reciprocal manner. Next to this, these three factors are influenced by heredity and other factors as lifestyle behaviors, environment and personal attributes. An important aspect is motivation to be(come) physically active, whereas it can indicate how self-determined a person is to perform the behavior [10]. These theoretical frameworks gave the input to test our hypothesis that more residents become physically active according to the Dutch Physical Activity Guidelines [11], and subsequently have higher scores on health related fitness tests and experience a higher health related quality of life. Next to this, we expect that people who dropped out have a lower motivation score and that motivation scores will increase after a period of being more physically active.

Therefore, we used an explorative longitudinal study to validate the rational for this study. This study will reveal the reached target group of CSCs and whether these participants become physically active, maintain these behaviors, or drop out, and, if they do maintain those behaviors, become healthier over a period of time. In this way, it provides new and relevant insights for policy evaluation due to the focus on PA behavior and health-related fitness of participants from PA programs of CSCs. This is complementary to previous policy evaluation that was focused on policy formulation and introduction [12].

## Materials and methods

This explorative longitudinal study is part of the larger project ‘Connecting Care, Sport and PA,’ which is a multiple case evaluation study, conducted in nine municipalities spread over the Netherlands with in total 14 CSCs, to get more insight in the CSC function [13]. The project consists of two trajectories. The first trajectory focuses on the intermediary target groups: CSCs and professionals active in primary care, sport, and PA who implement lifestyle programs. CSCs are expected to realize a connection between different sectors and to achieve and sustain collaboration between these sectors. The second trajectory, which provides the input for this study, focuses on health and PA behavior changes of participants of lifestyle programs. The target group: adults from the neighborhood who participate in lifestyle programs organized by professionals from the alliances of the first trajectory. This study was registered with the Dutch Trial Register (NTR4986) and has been approved by the Medical Ethical Review Committee: CMO Regio Arnhem-Nijmegen (file number 2013–492).

### Participants

Participant recruitment occurred between September 2014 and April 2016. A purposive sampling method was used: participants were recruited if they took part in a lifestyle intervention, sports activity, or PA whereby a CSC was involved. CSCs indicated when a new lifestyle intervention or activity started and whether it was possible to include participants in our study. After a year of inclusion, inclusion rates were lower than expected to reach 640 participants as calculated in our sample size calculation [13]. Therefore, we included three more CSCs; so, in total, 17 CSCs from 12 municipalities were included in our study. We made subdivisions for CSCs according to size of the municipality [14], implementation strategy of the CSC funding [15], and the recruitment strategy [16] of CSCs (Table 1). Size of municipality was based on number of residents. We distinguished three different approaches to the implementation of the CSC function: (1) CSCs working only from the PA sector (PA sector); (2) CSCs working from care, welfare, and PA organizations (integral approach); and (3) CSCs working from a partnership between primary care, welfare, and PA organizations (partnership). We distinguished three approaches to recruit participants to take part in an PA programs or activities: (1) public relations (PR) such as flyers, social media, and word of mouth; (2) a personal letter from a CSC sent to all addresses of those of a certain age group, obtained from the population register of a municipality; and (3) referral by a professional from primary care, welfare, or otherwise.

**Table 1:** Overview of subdivisions per CSC for municipality size, implementation strategy and recruitment strategy.

	Municipality Size (Number of Residents)			Implementation Strategy^a^			Recruitment Strategy^b^		
	<100,000	100,000–300,000	>300,000	PA sector	Integral approach	Partnership	Referral	PR	Letter
CSC 1			x			X	60%	40%	0%
CSC 2			X			X	25%	75%	0%
CSC 3			X			X	30%	70%	0%
CSC 4		X		X			8%	2%	90%
CSC 5	X			X			45%	55%	0%
CSC 6	X			X			5%	40%	55%
CSC 7		X		X			1%	77%	22%
CSC 8		X				X	75%	25%	0%
CSC 9		X		X			5%	95%	0%
CSC 10		X			X		10%	90%	0%
CSC 11			X		X		10%	90%	0%
CSC 12			X		X		Unknown	Unknown	Unknown
CSC 13		X			X		20%	80%	0%
CSC 14		X			X		Unknown	Unknown	0%
CSC 15^c^	X			Unknown	Unknown	Unknown	10%	90%	0%
CSC 16^c^		X		Unknown	Unknown	Unknown	100%	0%	0%
CSC 17^c^	X			Unknown	Unknown	Unknown	100%	0%	0%

CSC, Care Sport Connector; PA, physical activity; PR, public relations.

^a^ CSC funding was differently structural implemented. CSCs were imbedded in only the PA sector or an integral approach was adopted whereby CSCs were working either from care, welfare, or PA organizations, or in a partnership between primary care, welfare, and PA organizations.

^b^ Participants were recruited by a CSC in different ways to take part in the intervention or activity. Participants were approached by PR, such as flyers, social media, contact information of a CSC, and word of mouth, with personal letters, whereby the addresses were obtained from the population register of the municipality on the basis of age criteria, or were directed by a professional (primary care, welfare, and others) to take part in an intervention or activity.

^c^ CSCs are not included in the project ‘Care, Sport and Physical activity.’

#### Study design

We conducted a longitudinal study whereby participants conducted a fitness test and filled in a questionnaire at three time points (T_0_, T_1_, T_2_). T_0_ is before the start of the PA program or activity, T_1_ at the end of the PA program or after 6 months in case of a regular PA activity, and T_2_ 1 year after the start of the PA program or activity.

#### Outcome measures

Participants received a booklet with four questionnaires: the SQUASH [17]; the health-related quality of life questionnaire (Rand 36 [18]); motivation (Breq-2 [19]); and personal information (age, gender, somatic disorders, medication use, PA activity, and to which recruitment strategy they responded) questionnaires.

The PA level of a participant was measured with the Short QUestionnaire to ASsess Health-enhancing PA (SQUASH [17]). SQUASH measures the amount of PA, by asking the number of days and mean time spent per day per week, that participants carry out the following activities: commuting; PA at work or school; domestic work; and leisure time. Scores were converted according to the PA guidelines, and the PA variables were dichotomized, in order to indicate whether a participant meets the guideline of 30 minutes moderate to vigorous PA, at least five times a week (PA norm), is active on a vigorous intensity for 20 minutes at least three times a week (fit norm), meets at least one of these two norms (combi norm), and practices a PA activity (sport norm) [11]. Health-related quality of life was measured with the Rand 36 [18]. It measures functional status (physical functioning, social functioning, role limitation physical and emotional), wellness (mental health, vitality, and pain), and a general evaluation of health (general health, health change). Each score is transformed, so a higher score means a better health state, on a scale of 0–100 [18]. The Breq-2 questionnaire [19] was used to measure the continuum of self- determination to determine if a person is unmotivated or intrinsically motivated for PA. Each subscale (amotivation, external regulation, introjected regulation, identified regulation, and intrinsic regulation) will give a mean score. These means are multiplied with a weighting, which is set for each subscale, and these weighted scores are summed. A higher score indicate a higher degree to which a person feels self-determined to be physically active [19].

In addition, participants underwent a fitness test to test their health-related fitness at T_0,_ T_1_, and T_2_. The components of our fitness test are based on the Toronto Model [9], whereby health-related fitness is divided into the following five factors: morphological; muscular strength and endurance; motor fitness; cardio-respiratory fitness; and metabolic fitness. For each factor, suitable tests were selected based on their ability to assess functional fitness. The tests were not too demanding for older adults and people with (or with an increased risk of) a chronic disease and practically feasible to execute in any local situation. Morphological factors mass, height, fat percentage (measured using the Omron Body Fat Monitor BF306), and waist circumference were recorded to the nearest 0.1 kg, 0.1% or 0.5 cm. Mass and height were used to calculate body mass index (BMI). Shoulder, back, and leg flexibility was tested using the back-scratch test [20], the Modified Schober test [21], and the straight leg raise [22], respectively. For each test, participants made three attempts. For the Modified Schober test and straight leg raise test, the mean score was recorded. For the back-scratch test, the best attempt was recorded. The metabolic components blood pressure (measured while seated), cholesterol, and glucose were measured using the Omron Body Fat Monitor BF306 or Omron M5-1 and the Accutrend Plus meter, in line with the protocol of punctures [23]. Muscular strength and endurance were measured with the arm curl test [20], the chair stand test [20], and the hand-grip strength test [20]. For the latter, the JAMAR Hand Dynamometer was used, and participants had two attempts. Of the two attempts, the maximum score was noted. The up and go test [20] was used to test agility, speed, and dynamic balance for the motor functioning component. Scores were noted to the nearest 0.1 second. For the measurement of cardiorespiratory fitness, two tests were used, the 6-minute walk test [24] and the Astrand cycling test. The walk test was used for older adults (50+ years) and participants who were not used to cycling. In this way, we had two types of scores for cardiorespiratory fitness. We used norm equations [24, 25] to reveal the deviation from the norm for each participant as a percentage. Percentages from both norms were transformed to a z score, which gave us the same value for each test and made it possible to measure changes over time with one value for cardiorespiratory fitness.

All components of the functional fitness test were described in a protocol to make sure that each test leader performed the test in an identical way. Each test leader was instructed by ES (first author), and she was on site at 80% of the fitness tests. When she was absent, an instructed physiotherapist took over. After the fitness test, all participants received the questionnaire and had 3 weeks to fill in the questionnaire. A reminder was sent after 2 weeks. All participants provided informed consent and agreed to participate voluntarily.

### Statistical analysis

**Fig 1:**
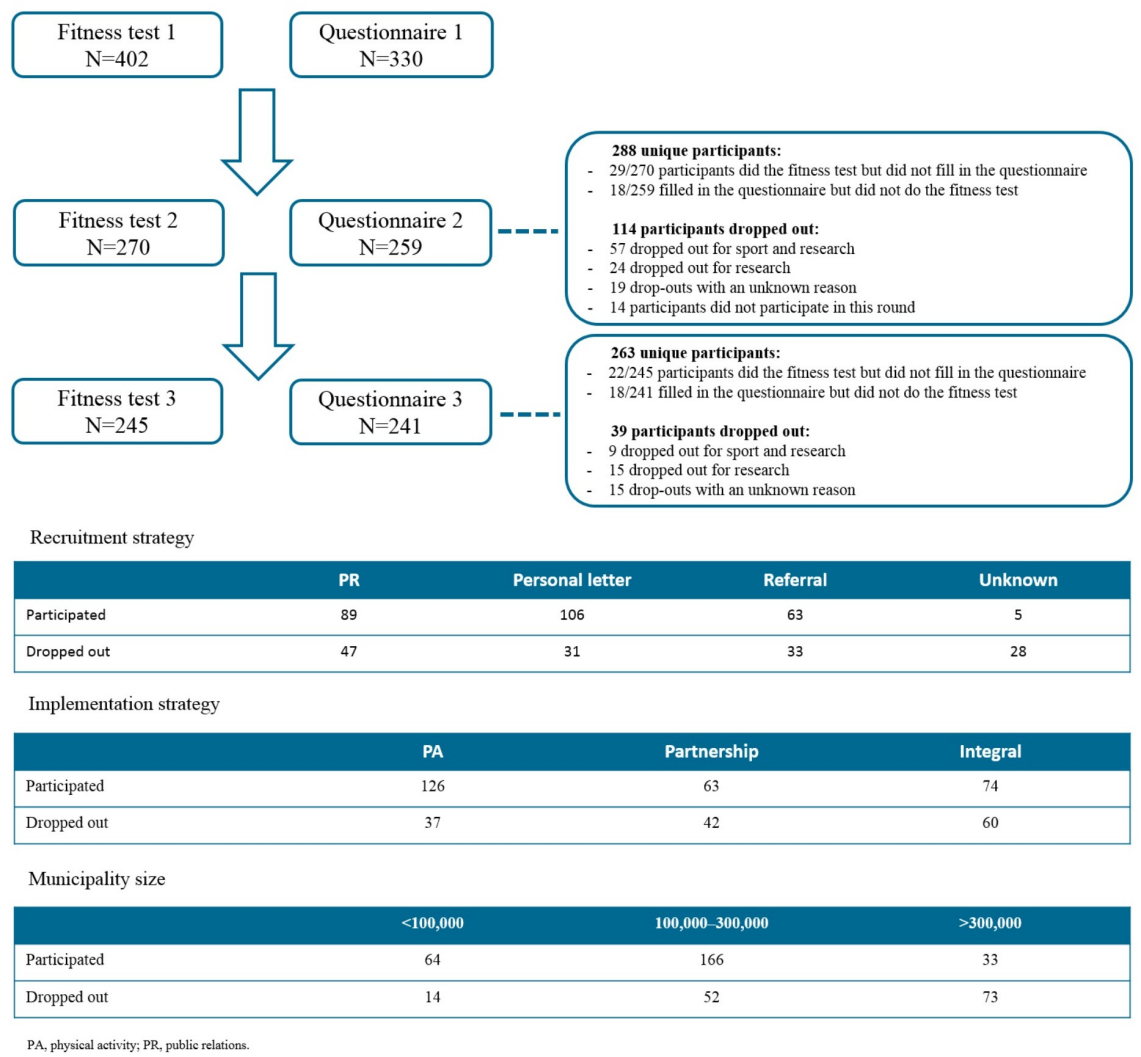
Overview of participation and drop-out rates.

First, we described the number of participants for each round (Fig 1). Second, we compared the group of those who dropped out to the group who continued to the end, for all norms and scores measured at the start (Table 2). For the comparisons we used a chi-square test in case of qualitatitive norms, and one-way ANOVA (equivalent to two-sample t-test) for the numerical scores. Third, we analyzed the (changes over the three moments of measurement in) scores of the maintainers. In Table 3 we present the measured means for the three time moments. Due to the hierarchical structure of our study, we used a multi-level (mixed model) analysis to test if average scores of participants differ over time. For all numerical performance scores, we used a mixed model with CSC (17 levels) and participant (nested in CSC) as random factors; approach, time, and recruitment strategy were used as fixed factors with time-approach and time-recruitment strategy interaction effects, while gender and age were used as covariates. For each variable we give the p-value for the F-test for differences in mean score between time-moments. Fisher’s protected LSD was used to determine the significance of the pairwise differences. Medication-related variables (i.e., blood pressure, cholesterol, and blood glucose level) were also adjusted for associated medication use. Fourthly, to establish differences in proportions of people fulfilling each of the four PA-level variables, we used a generalized linear mixed model (GLMM) with CSC and participant (nested in CSC) as random factors, with approach, time, and recruitment strategy as fixed factors including time-approach and time-recruitment strategy interaction effects. Municipality size is not tested as an interaction effect because these numbers were not representative due to drop-out rates of 100% for two CSCs caused by an activity stop. A P-value of <0.05 was considered to be statistically significant. Differences between the 3 pairs of means were judged on significance using the Tukey method. The first three analyses were performed using SPSS version 22, the fourth analysis using the GLMER function in R, with binomial as the distribution.

**Table 2:** Scores for physical activity behavior, health-related quality of life, motivation, and health-related fitness for drop-outs and remaining participants (means and standard deviation).

	Drop-outs	Remaining Participants	P-value^d^
Meets guideline n (%)^a^			
• PA norm	34 (40.9%)	143 (59.4%)	0.004
• Fit norm	17 (20.5%)	93 (38.6%)	0.003
• Combi norm	35 (42.3%)42 (50,6%)	156 (64.7%)	<0.001
• Sport norm		139 (57.7%)	0.263
Health-related quality of life (0–100)^b^			
• Physical functioning	69.8 ± 25.0	72.5 ± 22.9	0.374
• Social functioning	73.4 ± 23.3	80.6 ± 22.6	0.016
• Role limitation physical	67.5 ± 41.0	73.5 ± 38.5	0.248
• Role limitation emotional	69.7 ± 41.2	83.7 ± 32.4	0.003
• Mental health	68.8 ± 21.1	74.3 ± 17.4	0.020
• Vitality	55.6 ± 19.6	64.1 ± 19.4	<0.001
• Pain	70.4 ± 24.4	72.9 ± 24.3	0.423
• General health	57.8 ± 17.8	60.9 ± 17.5	0.168
• Health change	49.1 ± 21.0	48.2 ± 18.0	0.728
Motivation (–25–19)^b^	7.4 ± 5.0	8.6 ± 5.3	0.066
Number of somatic diseases^c^	1.21 ± 1.8	1.86 ± 1.8	<0.001
BMI (kg/m^2^)^c^	30.3 ± 6.6	29.6 ± 5.6	0.261
Waist circumference (cm)^c^	98.6 ± 16.2	99.5 ± 14.4	0.603
Fat percentage (%)^c^	38.1 ± 9.0	37.0 ± 8.5	0.225
Glucose (mmol/l)^c^	5.7 ± 1.5	5.8 ±1.7	0.420
Cholesterol (mmol/l)^c^	5.4 ± 0.9	5.6 ± 1.1	0.073
Blood pressure^c^			
• Diastolic (mmHg)	85.6 ± 11.0	85.3 ± 10.0	0.813
• Systolic (mmHg)	135.4 ± 21.9	140.1 ± 16.7	0.017
Shoulder flexibility (cm)	8.4 ±10.3	9.7 ± 10.0	0.250
Leg flexibility (degrees)	79.5 ± 13.5	79.8 ± 15.3	0.868
Back flexibility (cm)	20.7 ± 2.3	20.5 ± 1.9	0.214
Leg strength (n)	12.9 ± 3.7	12.8 ± 3.9	0.762
Arm strength (n)	15.8 ± 4.7	16.3 ± 5.1	0.330
Hand-grip strength (kg)	59.6 ± 19.5	61.4 ± 20.3	0.390
Speed, agility, and balance (second)^c^	6.9 ± 2.3	6.7 ± 2.1	0.450
Endurance (z)	–0.3 ± 1,0	0.1 ± 1.0	0.002

The data represent the means of each score with the corresponding standard deviation. BMI, body mass index; PA, physical activity.

^a^ PA norm is the guideline of 30 minutes of moderate to vigorous PA, at least five times a week. Fit norm is the guideline of being active on a vigorous intensity for 20 minutes at least three times a week. If someone meets at least one of these two norms, they meet the combination norm, and the sport norm is reached if a participant practices a PA activity [11].

^b^ Total possible score on the scale.

^c^ A lower score represents a higher level of fitness

^d^ For all qualitative norms, we used a chi-square test; for the numerical scores, one-way ANOVA (equivalent to two-sided two-sample t-test) was used.

**Table 3:** Scores of maintainers for physical activity behavior, health-related quality of life, motivation, and health-related fitness per measurement moment (means and standard deviation).

	T_0_	T_1_	T_2_	P-value
Meets guideline n (%)^a^				
• PA norm	143 (59.3%)^c^	158 (70.9%)^b^	159 (67.9%)^b^	0.007
• Fit norm	93 (38.6%)^c^	103 (46.2%)^b^	98 (41.9%)^b^^c^	0.028
• Combi norm	156 (64.7%)^c^	167 (74.9%)^b^	160 (68.4%)^b^^c^	0.009
• Sport norm	139 (57.7%)^c^	173 (77.6%)^b^	181 (77.4%)^b^	<0.001
Health-related quality of life (0–100)^d^				
• Physical functioning	72.5 ± 22.9	75.2 ± 22.1	75.1 ± 23.6	0.138
• Social functioning	80.6 ± 22.6	82.4 ± 20.6	81.9 ± 20.8	0.866
• Role limitation physical	73.5 ± 38.5^b^	77.2 ± 36.9^b^	70.6 ± 40.5^c^	0.018
• Role limitation emotional	83.7 ± 32.4	84.6 ± 32.6	80.8 ± 35.8	0.156
• Mental health	74.3 ± 17.4	76.6 ± 16.2	76.4 ± 16.9	0.254
• Vitality	64.1 ± 19.4	66.9 ± 18.9	65.3 ± 18.9	0.770
• Pain	72.9 ± 24.3	73.4 ± 24.5	74.4 ± 24.3	0.236
• General health	60.9 ± 17.5	62.3 ± 17.5	61.6 ± 17.6	0.478
• Health change	48.2 ± 18.0^b^	56.7 ± 20.2^c^	52.4 ± 20.2^e^	<0.001
Motivation (–25–19)^d^	8.6 ± 5.5^b^	9.8 ± 4.7^c^	9.8 ± 4.9^c^	0.123
Number of somatic diseases^f^	1.86 ± 1.77^b^	1.75 ± 1.80^c^	1.71 ± 1.76^b^^c^	0.016
BMI (kg/m^2^)^f^	29.6 ± 5.6 ^b^	29.6 ± 5.6^c^	29.5 ± 5.3^c^	0.011
Waist circumference (cm)^f^	99.5 ± 14.4^b^	99.3 ± 14.4^c^	98.7 ± 14.4^c^	<0.001
Fat percentage (%)^f^	37.0 ± 8.5	37.3 ± 8.6	37.4 ± 8.2	0.383
Glucose (mmol/l)^f^	5.8 ± 1.7	5.9 ± 2.2	6.0 ± 2.0	0.882
Cholesterol (mmol/l)^f^	5.6 ± 1.1	5.6 ± 1.1	5.6 ± 1.1.	0.343
Blood pressure^f^				
• Diastolic (mmHg)	85.3 ± 10.0^b^	84.5 ± 10.1 ^c^	83.5 ± 10.2^c^	0.016
• Systolic (mmHg)	140.1 ± 16.7^b^	137.7 ± 17.7^c^	136.7 ± 17.2^c^	<0.001
Shoulder flexibility (cm)	9.7 ± 10.0^b^	8.5 ± 9.6^c^	9.5 ± 9.9^c^	0.036
Leg flexibility (degrees)	79.8 ± 15.8^b^	81.7 ± 14.3^c^	83.3 ±14.4^e^	0.059
Back flexibility (cm)^f^	20.5 ± 1.9	20.6 ± 1.8	20.4 ± 1.7	0.322
Leg strength (n)	12.8 ± 3.9^b^	14.2 ± 4.0^c^	13.6 ± 4.0^c^	<0.001
Arm strength (n)	16.3 ± 5.14^b^	17.7 ± 5.1^c^	17.5 ± 5.4^c^	<0.001
Hand-grip strength (kg)	61.4 ± 20.3^b^	61.8 ± 18.8^c^	61.2 ± 18.8^b^^c^	0.005
Speed, agility, and balance (second)^f^	6.7 ± 2.1	7.0 ± 3.7	6.9 ± 1.6	0.596
Endurance (z)	0.1	0.0	0.0	0.906

The data represent the means of each score with the corresponding standard deviation. Scores were adjusted for age, gender, and medication for medication-related variables (blood pressure, glucose, cholesterol). BMI, body mass index; PA, physical activity.

^a^ PA norm is the guideline of 30 minutes of moderate to vigorous PA, at least five times a week. Fit norm is the guideline of being active on a vigorous activity for 20 minutes at least three times a week. If someone meets at least one of these two norms, they meet the combination norm, and the sport norm is reached if a participant practices a PA activity [11].

^b c e^ Represents whether there is a difference between scores over time.

^d^ Total possible score on the scale.

^f^ A lower score represents a higher level of fitness.

## Results

In total, 402 participants (75% were woman, and the mean age was 61.3 years [SD = 14.1]) from PA programs of CSCs were included in our study and underwent the first fitness test (Fig 1). Of them, 330 filled in the first questionnaire. Participants joined different PA programs or activities such as a combined lifestyle intervention, PA groups, aerobics, yoga, walking, running, dance, table tennis, cycling, and fitness. At T_1_, 270 participants completed the fitness test, of whom 241 filled in the questionnaire. There were 18 participants who filled in the questionnaire but had no score on the fitness test. In total, 114 participants dropped out at T_1_. Participants who dropped out were classified in four groups: participants who did not participate anymore in the PA activity and, as a consequence, did not participate in the study (n = 57); participants who did not want to participate in the study anymore (n = 24); participants without a known reason for drop-out (n = 19); and participants who were absent at T_2_ but present at T_3_ (n = 14). At T_2_, 245 participants completed the fitness test, of whom 223 filled in the questionnaire. There were 18 participants who filled in the questionnaire but had no score on the fitness test. Participants who dropped out (n = 39) were classified as follows: no participation in PA activity and study (n = 9); no participation in the study (n = 15); and unknown (n = 15). In total, 191 participants took part in three fitness tests and filled in all three booklets with questionnaires.

The purpose of CSCs is to stimulate residents who are inactive to become more physically active. Of 201 participants who filled in all three questionnaires, not many of them changed their PA levels. At T_0,_ 123 participants meet the PA norm. Of them, 104 participants stay active and the other 19 participants did not meet the PA norm at T_2_. The other 78 participants did not meet the PA norm at T_0_; of these, 38 participants met the norm at T_2_, and the remaining 40 participants still did not meet the PA norm at T_2_. This situation, wherein most people do not change in PA level, is also visible for the fit norm, combination norm, and sport norm. However, the fit norm was achieved the least, both at the start and after 1 year. It is noteworthy that the drop-out rate is larger for big municipalities, an integral approach, and partnerships. An overview of these participation rates is shown in Fig 1.

In total, 139 participants (76% were woman, and the mean age was 55.9 years [SD = 18.0]) dropped out from PA programs, whereas 263 participants (73% were woman, and the mean age was 63.6 years [SD = 12.0]) stayed active. In general, participants who dropped out were less physically active compared with the other participants on baseline. Differences are significant for the PA norm [X^2^(1) = 8.41, P = 0.004], fit norm [X^2^(1) = 9.03, P = 0.003], and combination norm [X^2^(1) = 12.99, P = 0.000], but not for the sport norm [X^2^(1) = 1.25, P = 0.263]. The items for health-related quality of life differ significantly for the mental, emotional, and vitality aspects, not for the physical and general aspects. The scores from the fitness test and motivation score showed no difference between the participants who dropped out and the other participants, except for number of somatic diseases (F = 11.827, P = 0.001), systolic blood pressure (F = 5.757, P = 0.017), and endurance (F = 10.129, P = 0.002), whereas participants who dropped out score less favorably for endurance. The chi-square test showed dependency of PA level and municipality size [X^2^(2) = 14.56, P = 0.001], approach [X^2^(2) = 22.89, P = 0.000], and recruitment strategy [X^2^(2) = 16.99, P = 0.000]. Bigger municipalities include more participants who are inactive, as do municipalities with an integral approach and CSCs who recruit participants by referral. Table 2 gives an overview for the scores of each group and the corresponding P-value.

The changes in scores for the maintainers over time are significantly different for the PA levels (Table 3). However, only the PA norm and sport norm still show increases after 1 year. There was an interaction effect of time with approach for the PA norm (P = 0.049), combi norm (P = 0.040), and sport norm (P = 0.040). A lower rate of participants met the requirements of the combi norm at the start of the PA program in municipalities with an integral approach, and this rate increased over time. There was no interaction effect of time with recruitment strategy.

The change of health-related fitness over time is minimal, but it is significant for blood pressure, BMI, waist circumference, shoulder flexibility, and leg and arm strength. Most of these variables show an improvement in time in the short term; however, after 1 year, scores stagnated or declined. For BMI, waist circumference, cholesterol, arm and grip strength, and agility, there was an interaction effect with recruitment strategy. The starting values were less favorable for the referred group compared with the PR and personal letter group; this difference remained the same over time for the three groups. The interaction effect of time with approach was significant for cholesterol, leg flexibility, back flexibility, and grip strength. The scores for participants from a municipality with the PA sector approach were higher on grip strength but lower for flexibility, whereas the progression over time did not show meaningful differences. An opposite trend regarding cholesterol is notable for the PA sector approach: their level rises at T_1_, whereas it decreases for the other approaches. See graphical representation in S1 Appendix.

The health-related quality of life components increased between T_0_ and T_1_, but decreased or stagnated between T_1_ and T_2_. Only physical role limitations, health change, and number of somatic diseases is significantly different over time. There is an interaction effect of time and recruitment strategy for physical functioning and general health perception. The referred group scores the least favorable at T_0_; however, at T_2_, similar results were observed between the three groups. Only health change and motivation showed a significant interaction effect between time and approach. The change in health increased the most for the integral approach from T_0_ to T_1_, at T_2_ scores are similar for all three approaches. See graphical representation in S1 Appendix.

## Discussion

The purpose of this study was to reveal whether participants of PA programs or activities of CSCs become physically active, maintain these behaviors or drop out, and become healthier. This study showed that approximately one-third of the participants dropped out. Participants who dropped out were less physically active than those who maintain activity and were more often reached in bigger municipalities, by an integral approach. However, almost 50% of the included maintaining participants who were not active at T_0_ became active at T_2_. The PA level changed over time: after 1 year, more participants met the PA guideline and sport norm. Despite the fact that this does not lead to meaningful gains in health-related physical fitness and quality of life, it is an important achievement that more people participate in sports and are more physically active.

The observed drop-out rate of this study was expected, as we know that 25–35% drop out from moderately active activities and 50% from vigorous exercise activities [26]. Unfortunately, participants who are less physically active drop out; however, they could benefit the most from PA and they are the intended target group of a CSC. Drop-out rates decrease with time, which could assume that participants adopt PA in their lifestyle if they were capable of starting PA in the first place. To realize the desired change in behavior, it is important that an individual is capable, has the opportunity, and is more motivated toward PA than sedentary behavior [27]. The current PA programs had a focus on PA itself but should also focus on possibilities to initiate a behavior change, by using, for example, the mechanisms of actions of the behavior change wheel [28].

Many interacting components are necessary to develop an intervention to change behavior, which makes it complex [29]. Looking at the minimal behavior change and health effects in this study, we can question whether a stand-alone PA program implemented by a CSC could reach the goal of getting people who are inactive to become more physically active and improve their health. Only a few PA programs of CSCs were highly intensive or offered in combination with a nutrition component. Previous studies [30, 31] revealed that the combination of PA and nutrition adaptation is more effective than a PA program alone, to reach health gains. Thereby, the intensity and frequency of a PA activity are crucial to reach health benefits. People should be active on a moderate activity level for 30 minutes five times a week and perform vigorous activity for 20 minutes three times a week [11]; a PA program should work toward this guideline. To create this behavior change, it is necessary to use different techniques tailored to the target group. Examples are social support, goal setting, reframing, self-monitoring, graded tasks, and restructuring the environment [32]. Conversation with CSCs during our project revealed that these techniques were missing in the offered local PA activities.

However, this effort of CSCs was not purposeless, starting with PA is the beginning of everything. When someone is getting used to a routine and is able to increase the frequency and intensity of PA, health results will follow. Only a small group of people can do this with a stand-alone PA activity. Our results show that the intended target group, i.e., people who are inactive and suffer from or have a high risk of developing a chronic disease, do not belong to this small group. But they were able, with this stand-alone PA program, to counteract a decline of their health-related fitness.

Nevertheless, another approach is necessary. Previous studies showed the effectiveness of combined lifestyle interventions with intensive guidance to create a change in behavior and health-related fitness [30, 31]. It would be helpful to involve primary care and welfare professionals in lifestyle interventions. They have the experience to guide people throughout a behavior change, give tailored information about the benefits of PA and in which way it should be added to their daily life, or to give tailored dietary advice. However, we have to take into account that a well-known pitfall is that transferal levels, from primary care to physical activities, often lag behind desired levels [33–35]. CSCs are expected to counteract this scenario, but our study showed that most PAs are offered by local sport or welfare organizations. Only a few activities offered intensive guidance, tailored information, and graded intensity. The advantage of local offerings is the continuity of the offerings, whereas interventions that are subsidized and offered in primary care have an end date. Our study showed that those local offerings helped participants to stay physically active, despite the fact that this did not result in health benefits. Still, we have to take into account that local providers do not have a framework—such as professional trainers, knowledge, finances, and sufficient participants—for new and other activities with a focus on behavior change and health promotion [36].

Therefore, CSCs should focus on uniting primary care and the PA sector to combine the strengths of both sectors. As we described in the introduction, this is their task. However, our study showed that both sectors are an extension of each other. Primary care professionals refer patients or help with the determination of health-related fitness, whereas the PA sector provides PA activities [14]. So there is a connection, more or less, but the results for health-related fitness are minimal. By uniting both sectors, we mean that combined lifestyle interventions are offered jointly. Primary care, PA, and welfare professionals design a program together, and this can be carried by each professional simultaneously. In this way, combined lifestyle interventions can be well designed by using all opportunities, knowledge, and strengths and offered by a suitable professional. Instead of placing the participant or referring them to a new activity or professional, participants are well informed at the start about involved professionals and the decrease or increase of elements in the combined lifestyle intervention. This is more than being linked or referred to each other and asks for an investment from all parties, including local, regional, and national policy.

### Strengths and weaknesses

This study is a practice-oriented study and a realistic representation of what happens in various municipalities of the Netherlands. However, this causes a weakness in evidence. The variety of offered PA activities ensures that we cannot decide whether a specific PA activity is proven effective. To do so, many more participants per municipality, recruitment strategy, approach, and PA activity would be required. However, this was not the goal of our study. There is plenty of evidence about the benefits of PA but not enough insight in how to offer PA and stimulate residents to become physically active in a local context. Our results helped local professionals by showing not only these results but also the results per CSC. Such results per CSC were not sufficient for a statistical analysis but gave an insight in their approach. This showed that the more frequent and intense PA resulted in more health benefits. Next to this, our study contained many variables, which could lead to significant results by chance. For some variables, this might be the case; however, again, this was not our goal, and we based our conclusion on the overall picture. This picture was stable for most variables and sometimes with a significant change; however, most of the time, this was a minimal, irrelevant change per group or time period. And on the other hand, we did not take all influencing variables into account. As mentioned in the Toronto model, for example lifestyle behaviors, the social and physical environment, and heredity are influencing PA, health related fitness and health. It would be interesting to focus on these elements in further studies or to combine the results of multiple studies to get a more complete picture.

Another point to notice is our analysis regarding participants who dropped out and remaining participants. Participants dropped out from our research, the PA activity, or both, and for some participants, the reason was unknown. We decided to analyze the total group of participants who dropped out because we think that dropping out from our research is closely related to dropping out of the PA activity. This thought is based on the fact that the fitness test of our research was combined with the PA activity, which means we visit the PA activity to carry out the fitness test. However, this will not apply to the total group of participants who dropped out; still, if we analyzed a selected group of drop-outs, we had a similar problem. In our opinion, the group chosen for study here would lead to the most realistic picture, although someone else could come to a different choice. Next to this, our drop-out rate was as expected in our sample size calculation. Despite we added extra CSCs, we did not reach our inclusion rates of 640 participants, which resulted in an insufficient number of independent observations. However this data is valuable, which made us decided to analyze and publish our results.

## Conclusion

This explorative study showed that, overall, more people get physically active but only a few people improve their PA level. There are more people who stopped their PA after 1 year than people who became physically active and maintained this behavior. In addition, people who were less active dropped out and there was no meaningful gain in health-related fitness. It is necessary to invest in combined lifestyle interventions offered by a collaboration of primary care, welfare, and PA professionals. Lifestyle interventions should be offered with a higher frequency, intensity, and focus on behavior change with various techniques. CSCs need to invest in the unity of these sectors and direct professionals of these sectors to offer combined lifestyle interventions jointly. Local, regional and national policy should be supportive for this. Future studies should reveal which combination of preconditions are helpful to prevent drop-out or stimulate drop-out and whether jointly offered lifestyle interventions achieve better results regarding health-related physical fitness.

## Supporting information

S1 Appendix Graphical representation of interaction effects in these study. (PDF)
