# Supplementary material for: Do participants in a physical activity program from a Care Sport Connector become healthier? An explorative study from the Netherlands
Source: PLoS One. 2023 Dec 14;18(12):e0287913. doi: 10.1371/journal.pone.0287913 (PMC10721037; doi:10.1371/journal.pone.0287913)

Graphical representation of interaction effects in these study

Significant interaction effects of time * recruitment strategy

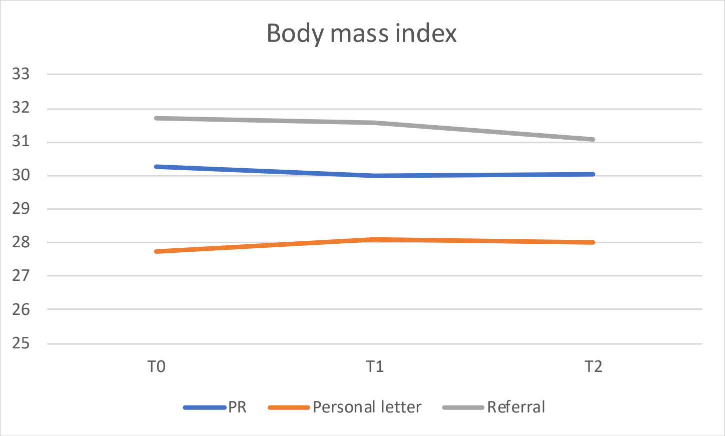

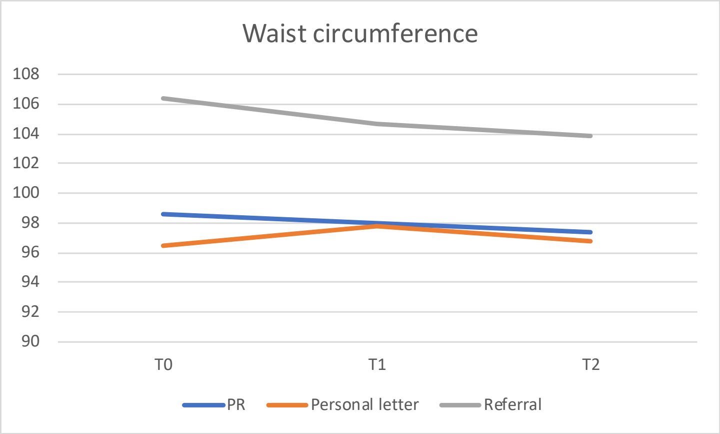

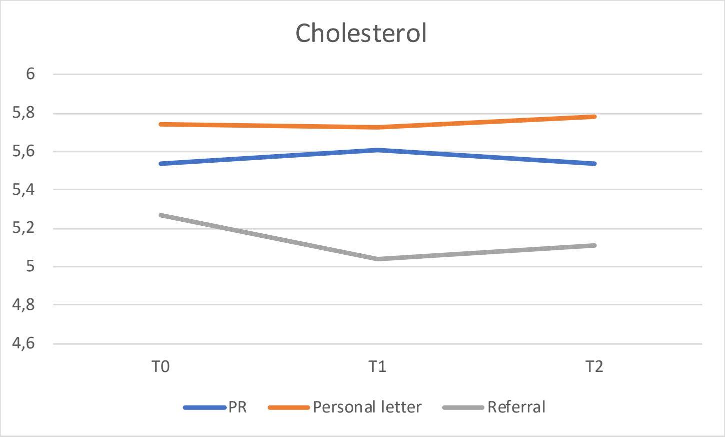

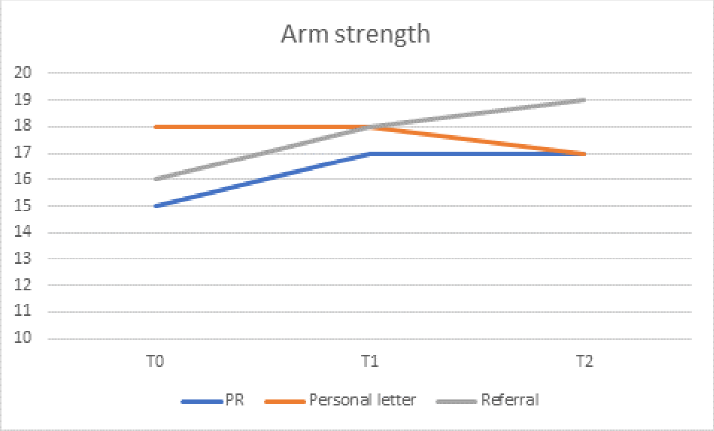

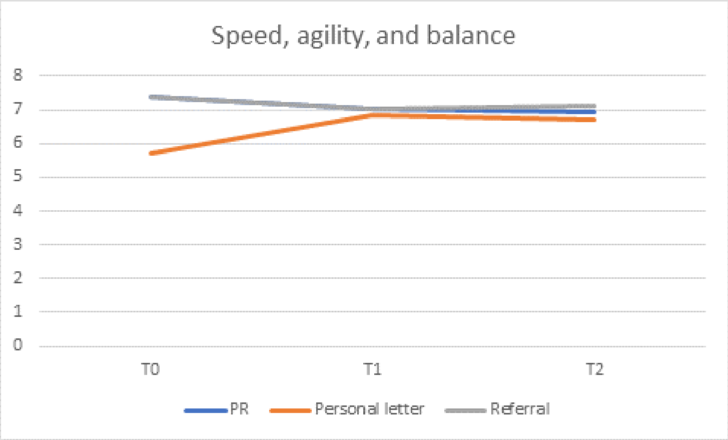

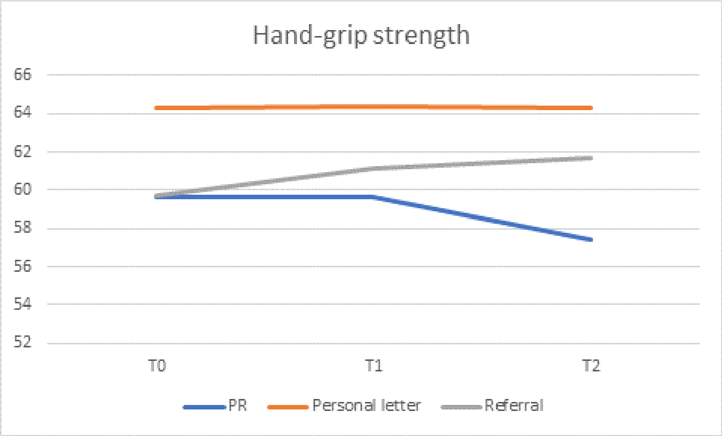

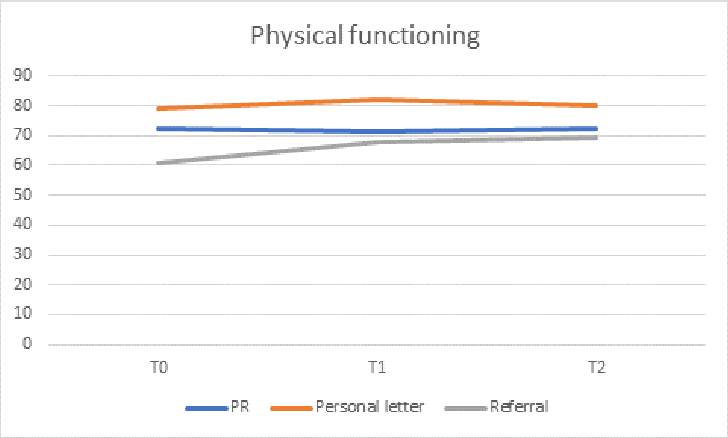

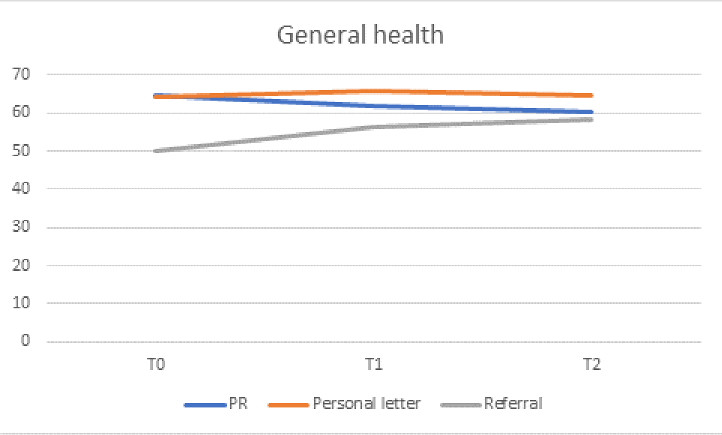

Significant interaction effects of time * approach

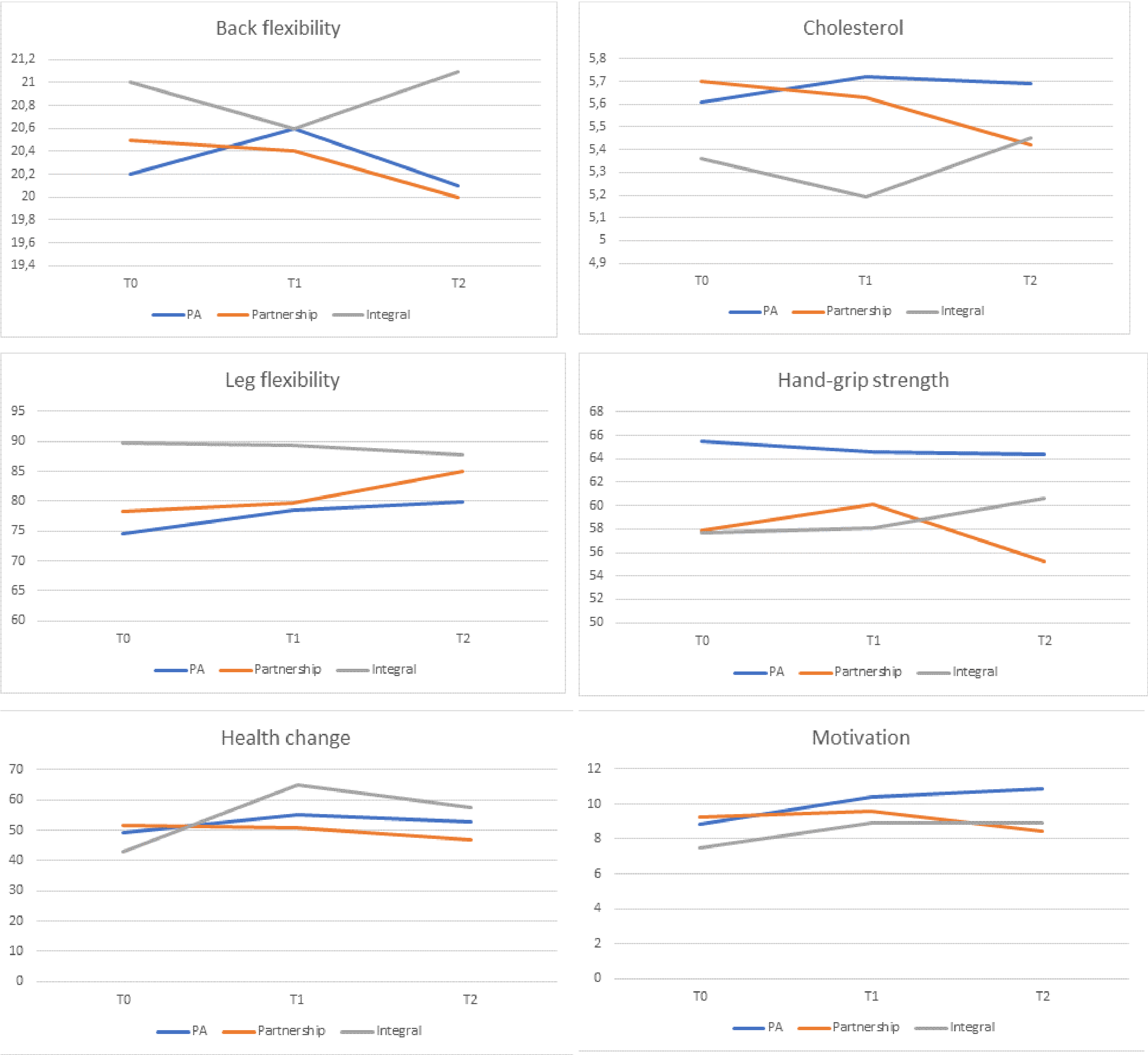

Supplement: S1 Appendix — (PDF) [file pone.0287913.s001.pdf]
